# Editorial: Situating phenomenological psychopathology: subjective experience within the world

**DOI:** 10.3389/fpsyg.2023.1204174

**Published:** 2023-08-25

**Authors:** Elizabeth Pienkos, Jérôme Englebert, Jasper Feyaerts, Rosa Ritunnano, Louis Sass

**Affiliations:** ^1^Department of Psychology, Duquesne University, Pittsburgh, PA, United States; ^2^Centre de recherche interdisciplinaire sur la déviance et la pénalité, Université Catholique de Louvain, Louvain-la-Neuve, Walloon Brabant, Belgium; ^3^Centre de recherches pénalité, sécurité et déviances (CRPSD), Université Libre de Bruxelles, Brussels, Belgium; ^4^Department of Psychoanalysis and Clinical Consulting, Faculty of Psychology and Educational Sciences, Ghent University, Ghent, East Flanders, Belgium; ^5^Centre for Youth Mental Health, Faculty of Medicine, Dentistry and Health Sciences, University of Melbourne, Parkville, VIC, Australia; ^6^Institute for Mental Health, School of Psychology, University of Birmingham, Birmingham, United Kingdom; ^7^Graduate School of Applied and Professional Psychology, Rutgers, The State University of New Jersey, Piscataway, NJ, United States

**Keywords:** phenomenology, phenomenological psychopathology, lived world, interpersonal, situated, contextual

## Introduction

The field of phenomenological psychopathology was originally developed in the early 20^th^ century. It has seen a recent resurgence, largely heralded by Sass and Parnas's (2003) influential ipseity disturbance model of schizophrenia. Throughout its history, this field has represented an important counter-trend to biomedical approaches to psychiatry, placing subjectivity at the forefront of psychopathological research (Parnas et al., 2013).

Several recent works have indicated the beginning of a shift in the emphasis of phenomenological psychopathology, broadening the view of subjectivity as extended and embedded in a social, cultural, and historical world. Such a move may be found in expanded models of schizophrenia (e.g., Sass et al., 2018; Englebert, 2021), attention to the personal and cultural meanings of symptoms (e.g., Feyaerts et al., 2021; Ritunnano, 2022), and greater focus on life historical and cultural events in phenomenological research (e.g., Van Duppen and Feyaerts, 2021; Pienkos, 2022).

With this Research Topic, we hope to mark the beginning of a ***situated phenomenological*** ***psychopathology***—an approach to studying mental disorders that explicitly recognizes the intertwining of self and world in subjective experience. The papers that have been included here reflect the current state of research and simultaneously point toward possibilities for future inquiry. They focus on a variety of mental or emotional disorders, including depressive disorders, body dysmorphic disorder, autism spectrum disorder, substance misuse, and contemporary manifestations of “hysteria,” as well as schizophrenia. We view the authors as raising and responding to several important questions that indicate the way forward for this situated phenomenology, which we summarize here.

## What is the relationship between psychiatric disorders and experiences of the external world?

Osler adds important nuance to characterizations of interpersonal experience in depression, finding that difficulties in shared “we-experiences” heighten the sense of aloneness, alienation, and difference in depressed individuals. Lindhardt et al. offer novel empirical data on the psychopathology of youth not in education, employment or training, finding that this population has been critically overlooked and is a candidate for prevention and early intervention strategies in schizophrenia-spectrum disorders. A previously unpublished case study by Erwin Straus, originally written in 1946 and introduced here by Moskalewicz and Fuchs, describes transformations of lived time and space in a case of severe depression, and how these are meaningfully linked with the patient's felt relationship to the world. These papers develop our understanding of the circular relationship between an individual and their interpersonal and practical world, and offer a glimpse of how this world of social and pragmatic interchanges may be engaged to modify the experiences of people with depression or schizophrenia.

## How are our diagnostic categories and criteria embedded in the world?

Mancini et al. argue that the diagnostic categories associated with “hysteria” are replete with historical and cultural assumptions, even in the contemporary manifestations of histrionic personality disorder and conversion disorder. This has had the effect of essentializing contingent symptoms of hysteria, historically resulting in over-diagnosis in women and an over-emphasis on sexuality. To remedy this, the authors advocate for searching for a homogeneous subjective core that is understood in the context of patients' lifeworlds, one that seeks recognition and the establishment of selfhood via the gaze of the other. Such a critique might push us to question the cultural contingencies of other disorders, including symptom manifestations, diagnostic criteria, and conceptualizations.

## What paradigms and methods are best suited to explore the relationship between self and world in psychopathology?

Boldsen uses phenomenological and ethnographic methods to explore the overwhelming and unpredictable sensory dimensions of interpersonal experience in autism. She finds that what are often viewed as pathological behaviors instead represent important “styles of being” that enable people with autism to navigate their worlds. Messas and Fernandez note that while phenomenological psychopathology has been fruitfully applied to understanding and explaining schizophrenia, the field's shift to incorporate the context of illness should also involve a shift in paradigm case. The particular relevance of the social and cultural context in which substance misuse occurs make it especially apt for demonstrating the value and methods of a contextual or situated phenomenology.

## How do world events impact the progression and amelioration of psychopathology?

Craythorne et al. use Interpretative Phenomenological Analysis to give voice to the often-neglected perspective of those living with Body Dysmorphic Disorder. They locate the roots of the disorder within enduring experiences of inter-personal rejection, shame and self-other discrepancy. Medford and Sigala's case study of a Huntington's patient with delusions of lycanthropy indicate the importance of understanding and addressing a patient's life history in the development of anomalous expressions of distress, even in illnesses that have entirely neurological origins. Irarrázaval and Kalawski investigate the role of emotional disruption in the development of psychopathology, finding that the empathic communication of psychotherapy can alter emotional experiencing and result in increased capacity for empathy. Vescey et al.'s study employs participatory action research to explore member experiences of a psychosocial clubhouse, showing how the recovery process is facilitated through enhancing agency and meaningful activity, and indicating that participatory methods can challenge assumptions embedded in models of psychopathology. These papers find that interactions in the world are not only impacted by psychopathology, but are also indispensable for understanding the development of psychopathology and recovery from it.

## Conclusions

These articles forcefully respond to the Research Topic while also opening up new lines of applied phenomenological research. We see the need for a different kind of sensitivity to the worldly context or situation in which psychopathology is embedded, requiring methodological development, expansion and cross-fertilization of disciplines. Researchers are called to situate their enquiries within particular, historically-grounded, and contextually-bounded lifeworlds. This collection also illustrates how attention to the environment may lead to a partial reconsideration of the psychopathological experience in a less strictly maladaptive or abnormal framework, but as the creation of new norms of existence in interaction within the world. If this approach is to take hold, it will be through the commitment of researchers to asking contextually-sensitive questions, attending to and modifying the limitations of current research methods, and remaining open to the novel and transformative insights that emerge from their work.

## Author contributions

EP prepared the main manuscript. JE, JF, RR, and LS provided additional content and editing. All authors contributed to the article and approved the submitted version.
